# Lid wiper epitheliopathy: an early sign of dry eye diagnosis

**DOI:** 10.3389/fmed.2025.1593430

**Published:** 2025-06-05

**Authors:** Yuan Gao, Meiting Huang, Wenjing Song, Yingsi Li, Xiaoming Yan

**Affiliations:** Department of Ophthalmology, Peking University First Hospital, Beijing, China

**Keywords:** dry eye, lid wiper, lid wiper epitheliopathy, partial blinking rate, lipid layer thickness

## Abstract

**Objectives:**

The purpose of this study was to investigate the relationship between the severity of lid wiper epitheliopathy (LWE) and ocular surface features and evaluate the potential of LWE as an early diagnosis indicator of dry eye.

**Methods:**

Eighty-eight patients diagnosed with dry eye by TFOS DEWS II were divided into two groups based on the Korb grading: the mild group and the moderate–severe group. Ocular assessments included examination of LWE, tear-film lipid layer thickness (LLT) measurement, partial blinking rate (PBR) calculation, fluorescein tear breakup time (FTBUT) measurement, determining corneal fluorescein staining score, eyelid margin score, and meiboscore.

**Results:**

In patients with upper LWE, the PBR and ocular surface disease index (OSDI) score were higher and LLT was lower in the moderate–severe LWE group (*p* < 0.05). In patients with lower LWE, the PBR and lower eyelid margin score were significantly higher in the moderate–severe LWE group (*p* < 0.05). The upper LWE staining score was moderately and significantly associated with the lower LWE staining score. Compared with LWE, if the FTBUT was used as the diagnostic indicator according to TFOS DEWS II, China, or ADES, the missed diagnosis rate fluctuated from 5.7 to 54.5%.

**Conclusion:**

The severity of LWE is related to dry eye indicators such as the PBR, FTBUT, eyelid margin score, OSDI, and meiboscore. Both upper and lower LWE can be used as diagnostic criteria for dry eye. Moreover, compared with FTBUT, LWE is more suitable as an early sign of dry eye diagnosis.

## 1 Introduction

**Figure 1:**
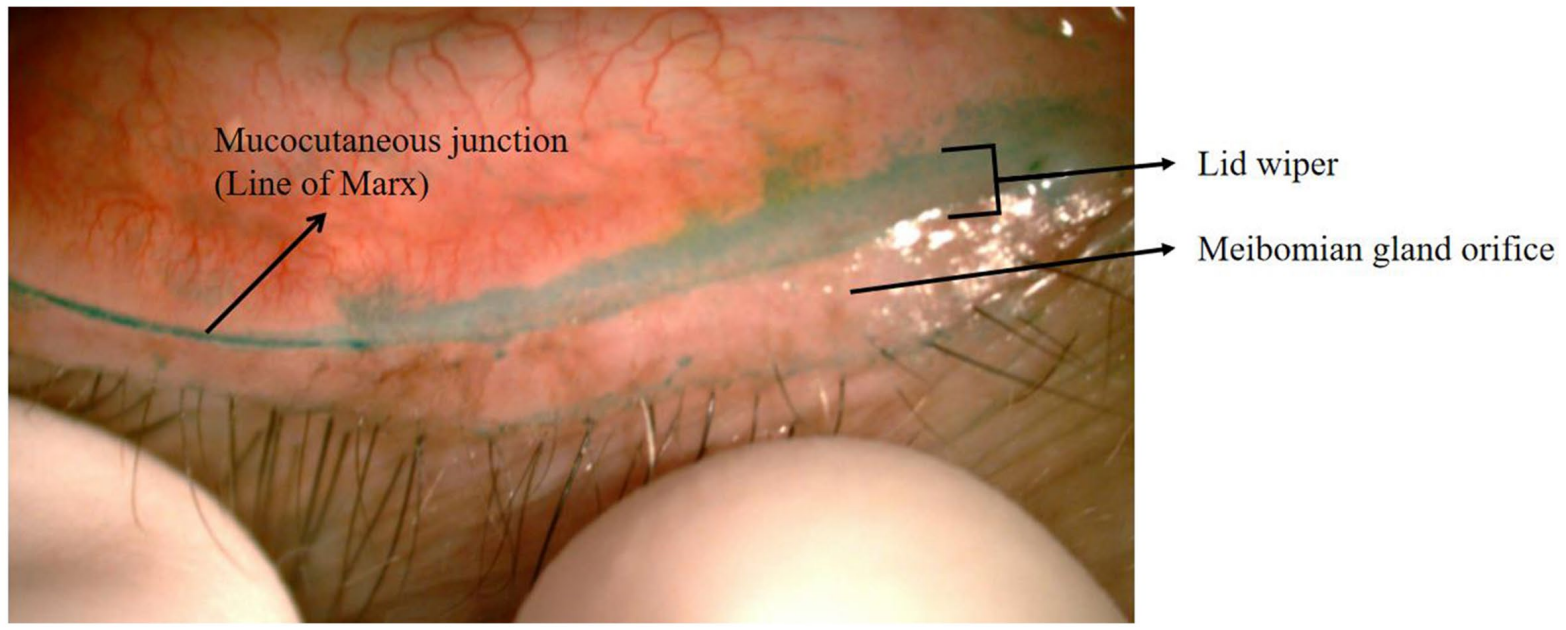
Eyelid of LWE with lissamine green staining. The location of the lid wiper and the line of Marx are shown here.

Korb et al. (1) first introduced the concept of “lid wiper epitheliopathy (LWE)” (Figure 1) in 2002, claiming that this lesion could be a precursor to dry eye. The fundamental explanation for the pathogenesis of LWE is increased friction between the lid wiper and the ocular surface as a result of inadequate lubrication. This led to the hypothesis that lid pressure, tear composition, tear viscosity, surface texture, and blink velocity could all play a role in LWE formation (1–3). Currently, our study focused on the effects of tear-film lipid layer thickness and the partial blinking rate on LWE.

The lipid layer is located in the tear film’s outermost layer, which appears to serve an important function in respreading the tear film and slowing the aqueous component’s evaporation following blinking (4). However, far too little attention has been given to the relationship between quantitative LLT measurement and the severity of LWE.

Apart from proper tear quantity and quality, complete blinking is a protective mechanism for the cornea and conjunctiva, which is required for ocular surface moisture, adequate reservoirs of secretion by meibomian glands, and tear lipid spreading (5–7). Accordingly, LWE may appear to be accelerated by mechanical damage caused by incomplete blink. However, there were only a few clinical trials that analyzed the relationship between PBR and LWE.

Furthermore, the majority of LWE investigations were based on scoring by grading the upper LWE. It has been suggested that there was a difference between upper and lower LWEs. According to some researchers, lesions in lower LWE were more severe owing to hyperosmotic insult and horizontal nasal ward movement, while others claimed that lesions in upper LWE were more severe because of the upper eyelid’s considerable vertical movement (8–10).

LWE was seen in 76% of patients with dry eye symptoms, compared to 12% of asymptomatic controls. LWE might explain the discomfort of these patients categorized as “pain without stain,” with no evidence of corneal staining or short fluorescein tear breakup time (FTBUT) (3). Our previous studies have also shown that LWE often accompanied dry eye symptoms even in cases whose other clinical signs did not support a dry eye diagnosis (11).

In this study, we stained, observed, and graded the upper and lower lid wiper regions of patients diagnosed with dry eye by TFOS DEWS II.

There were three aims in this study: (1) to investigate the relationship between the severity of LWE and ocular surface features such as LLT and partial blinking, (2) to assess the consistency and correlation between upper and lower LWE, and (3) to evaluate the potential of LWE as an early diagnosis of dry eye.

## 2 Methods

### 2.1 Study population

We recruited 88 patients diagnosed with dry eye by TFOS DEWS II at the Ophthalmology Department of Peking University First Hospital between March 2021 and December 2021.

The inclusion criteria included (1) patients aged between 20 and 80 years, (2) patients with OSDI scores ≥13, and (3) patients with FTBUT < 10s or with ocular surface staining (> 5 corneal spots, >9 conjunctival spots, or with LWE) according to TFOS DEWS II.

We excluded (1) patients with ocular infection or inflammation; (2) those using local or systemic antibiotics due to eye infection; (3) those using eye drops other than artificial tears in 6 months, (4) those who had used artificial tears within 4 h before the examination; (5) those with a history of ocular trauma or surgery; (6) those who had engaged in swimming, sauna activities, instillation of eye drops, or application of eye makeup 24 h before the examination; and (7) those with a history of wearing contact lenses within the last year.

All participants signed a written informed consent form that included a detailed summary of the study’s objectives, risks, benefits, and procedures. The Ethics Committee of Peking University First Hospital authorized this study (Approval no. 2021-468). Data from the left eye were used for analysis.

### 2.2 Grouping

According to the Korb grading method (1), the patients were divided into either the mild LWE group or the moderate–severe LWE group. All groups underwent the following ophthalmic examinations in order.

### 2.3 Dry eye questionnaire

OSDI (12): There are 0–100 points on the OSDI scale, and higher scores indicate more severe symptoms.

### 2.4 LLT measurement and PBR

The LLT was evaluated with a LipiView I Ocular Surface Interferometer (Johnson & Johnson Vision Care Inc., Santa Ana, CA). The LLT and PBR (number of partial blinks/number of total blinks) were recorded in 10 s. To assure the correctness of the data, a credibility (conformance factor, CF) >0.80 was necessary. Finis et al. (13) reported that an LLT < 60 nm indicated a 90% probability of meibomian gland dysfunction, so we divided the lipid layer thickness into <60 nm and ≥60 nm.

### 2.5 Eyelid margin score

**Figure 2:**
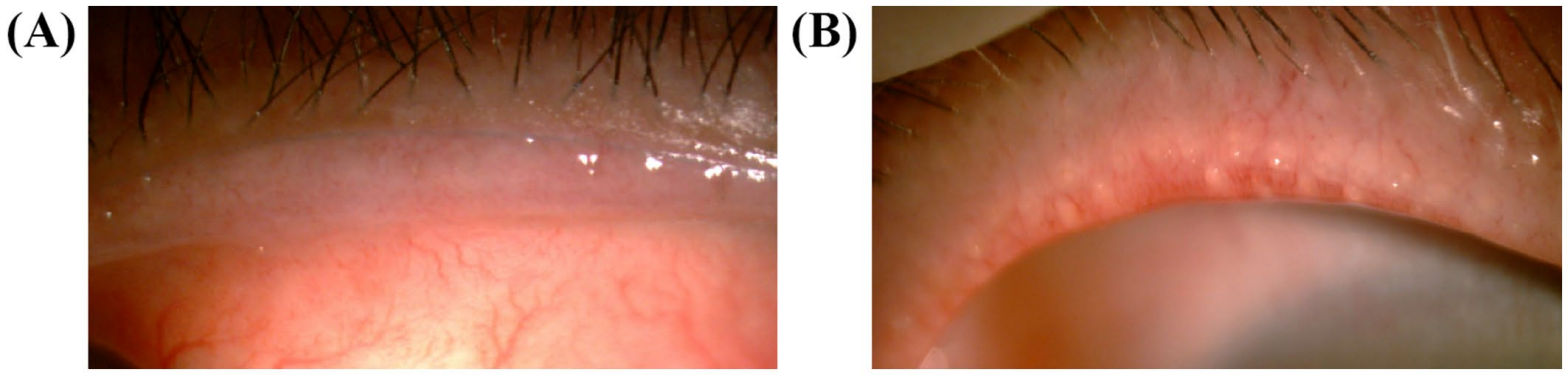
Photography of the eyelids. **(A)** The anterior positioning of the mucocutaneous junction and vascular engorgement were shown in a patient who was scored 2. **(B)** The vascular engorgement and plugged meibomian gland orifices in a patient who was scored 4.

The eyelid margin of the patients was observed under a slit lamp, and the eyelid margin morphology was graded according to the criteria of the International Workshop on Meibomian Gland Dysfunction (14). The morphology of the eyelid margin was graded using four criteria: irregular lid margin, vascular engorgement, plugged meibomian gland orifices, and anterior positioning of the mucocutaneous junction (Figure 2). If any of the above changes did not occur, the applicable score was recorded as 0; if any of the changes did occur, the applicable score was recorded as 1, and the overall score was recorded as 0–4.

### 2.6 FTBUT

Strips of fluorescein sodium (containing 1.0 mg fluorescein sodium) (Jing Ming New Technological Development Co., Ltd., Tianjin, China), instilled by approximately 5 μL (a drop) of normal saline, were used. The FTBUT was estimated by calculating the mean of three successive FTBUTs as measured by a stopwatch (15).

### 2.7 Corneal fluorescein staining

The examiner observed whether the corneal epithelium was stained under a slit lamp. The cornea was divided into four quadrants by a central cross. Each quadrant was allotted 0–3 points, and the total score was 0–12 points: 0 indicated non-coloring, 1 indicated 1–30 dot-coloring, 2 indicated more than 30 dot-coloring but no fusion, and 3 indicated dot-coloring fusion, filaments, and ulcers (16).

### 2.8 Examination and grading of the lid wiper region by staining

A lissamine green strip (Tianjin Jingming Electron Material, China) was soaked in normal saline and dropped into the inferior fornix conjunctiva; this step was repeated after 1 min. After 3 min, the wiper region of the upper and lower eyelids was observed. We applied the grading method of Korb to record and grade the level of lissamine green staining and the sagittal width of the upper and lower lid wiper region (Table 1). The final score for each patient was obtained by averaging the lissamine green staining grades. Classification was as follows: 0.25–1.0, graded 1 (mild LWE); 1.25–2.0, graded 2 (moderate LWE); and 2.25–3.0, graded 3 (severe LWE) (3).

**Table 1:** Grading of lissamine green staining of the lid wiper.

Staining of the lid wiper	Grade
Sagittal width of staining	
<25% of the width of the wiper	0
25–50% of the width of the wiper	1
50–75% of the width of the wiper	2
≥75% of the width of the wiper	3
Horizontal length of staining	
<2 mm	0
2–4 mm	1
5–9 mm	2
>10 mm	3

### 2.9 Meibomian gland dropout (meiboscore)

**Figure 3:**
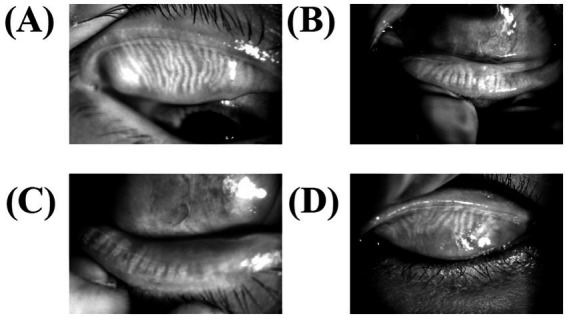
Meibography for meibomian gland dysfunction. The lower and upper eyelids were turned over, and MGs were observed using an infrared transmitting filter. **(A)** The upper meibomian gland deficiency of less than one-third (level 1), **(B)** the lower meibomian gland deficiency of less than one-third (level 1), **(C)** meibomian gland efficiency of more than two-thirds (level 3), and **(D)** deficiency between one-third and two-thirds (level 2).

The meibomian gland was exposed to the subjects’ upper and lower eyelids, and photographs of the gland were taken to document the deletion of the meibomian gland. Meibomian gland dropout was graded (Figure 3) according to the criteria of Arita et al. (17). The upper and lower eyelid scores were added to the final score.

### 2.10 Statistical analysis

SPSS for Windows was used for statistical analysis (version 26.0 SPSS). To determine the normality of each measurement index in each group, the Kolmogorov–Smirnov test was applied. Non-parametric tests were used since most of the variables did not have a normal distribution.

Indexes with a normal distribution were reported as the mean ± standard deviation, whereas those with a non-normal distribution or unequal variance were expressed as the median and quartile [M (Q1, Q3)]. The frequency for categorical data and the median (range) for continuous data were analyzed to compare categorical variables. The chi-square test was used, while the Mann–Whitney *U*-test was used to compare groups for numeric variables. Spearman’s correlation analysis was used to examine the relationships between the parameters (Spearman’s partial correlation analysis was calculated for LLT and LWE, adjusting for the effect of sex and age). The same researcher scored the upper and lower LWE, respectively, so we used Spearman’s correlation analysis to determine the consistency and relationships of the upper and lower LWE results. A *p* < 0.05 was considered statistically significant.

## 3 Results

### 3.1 Demographic characteristics

A total of 88 patients (88 eyes) aged 54.00 (32.25, 65.00) years were included in this study (35 males and 53 females). Sex (*p* = 0.075 and 0.060, respectively) and age (*p* = 0.592 and 0.651, respectively) were well balanced between the mild group and the moderate–severe group with upper LWE or lower LWE.

### 3.2 Factors that influenced the severity of LWE

Table 2 summarizes the LLT, PBR, FTBUT, corneal fluorescein staining, OSDI questionnaire, and lid margin score in patients with upper and lower LWE. According to Spearman’s analysis, the upper LWE staining score was weakly but significantly correlated with the OSDI score (r = 0.234, *p <* 0.05) and FTBUT (r = −0.216, *p <* 0.05). Meanwhile, the LLT and PBR showed no significant correlation with the upper LWE (r = −0.196, *p* = 0.071 and r = 0.158, *p* = 0.141, respectively). The lower LWE staining score was weak but significantly associated with the lower eyelid margin score (r = 0.287, *p* < 0.01) and the PBR (r = 0.237, *p* < 0.05). However, the LLT showed no significant correlation with the lower LWE (r = −0.088, *p* = 0.418). In addition, the LWE staining score of the left eye was weakly but significantly correlated with the meiboscore (r = 0.351, *p* < 0.01).

**Table 2:** Comparison of factors between the two groups with upper and lower LWE.

Ocular assessments	Upper-lid-wiper epitheliopathy		*p*-value	Lower lid wiper epitheliopathy		*p*-value
	Mild	Moderate–severe		Mild	Moderate–severe	
OSDI	31.25 (22.92,45.31)	41.67 (26.04,56.25)	0.040*	35.47 (25.00,52.08)	33.33 (25.00,52.08)	0.886
LLT, nm	60.00 (47.00,89.50)	48.50 (35.00,67.75)	0.046*	59.00 (42.00,81.50)	51.00 (35.00,70.00)	0.262
<60 nm (*n*, %)	21 (52.5)	30 (62.5)	0.344	21 (56.8)	30 (58.8)	0.846
60–100 nm (*n*, %)	19 (47.5)	18 (37.5)		16 (43.2)	21 (41.2)	
PBR (%)	50 (33.33,100.00)	80.00 (50.00,100.00)	0.046*	50.00 (20.00,100.00)	80.00 (50.00,100.00)	0.016*
Eyelid margin score (*n*, %)			0.101			0.027*
1	5 (12.5)	4 (8.3)		12 (32.4)	7 (13.7)	
2	13 (32.5)	7 (14.6)		17 (45.9)	24 (47.1)	
3	11 (27.5)	20 (41.7)		5 (13.5)	14 (27.5)	
4	11 (27.5)	17 (35.4)		3 (8.1)	6 (11.8)	
FTBUT(s)	5.51 (4.09, 6.39)	4.87 (3.04, 6.13)	0.114	5.24 (3.60,6.37)	5.07 (3.32,6.20)	0.594
Corneal fluorescein staining (*n*, %)			0.231			0.525
0	29 (72.5)	27 (56.3)		25 (67.6)	31 (60.8)	
1	6 (15.0)	17 (35.4)		9 (24.3)	14 (27.5)	
2	3 (7.5)	4 (8.3)		1 (2.7)	6 (11.8)	
3	2 (5.0)	0 (0)		2 (5.4)	0 (0)	

*p < 0.05.

### 3.3 Correlation for consistency of upper and lower LWE scores

**Figure 4:**
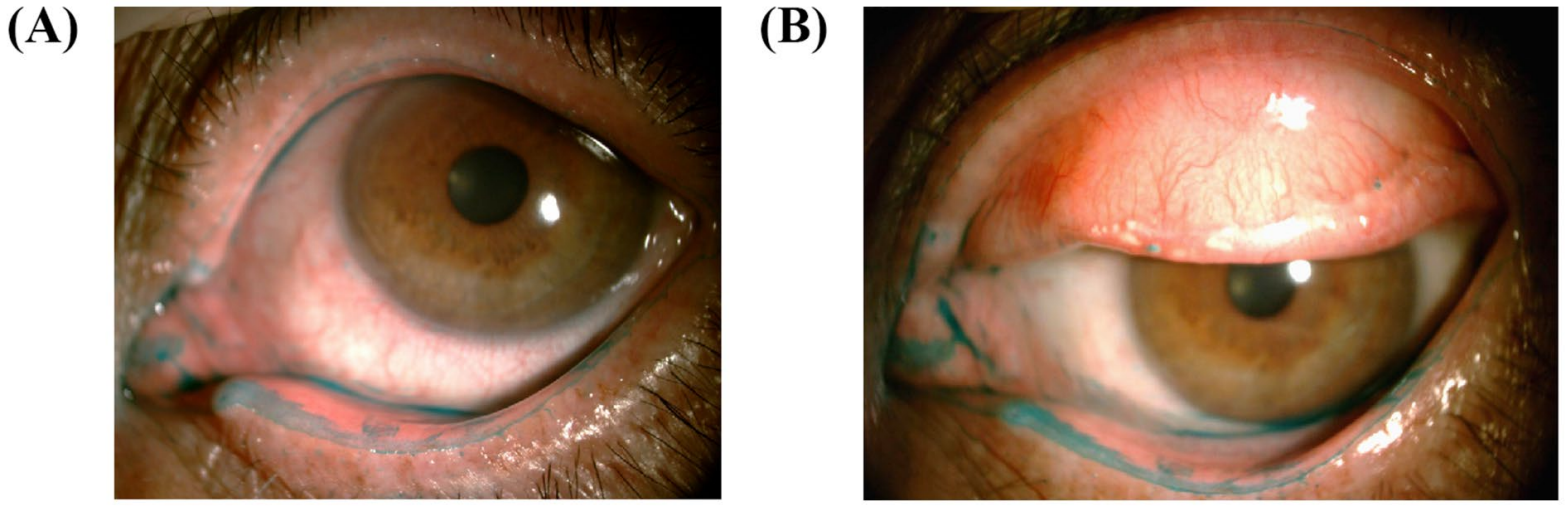
Images of upper and lower LWE are taken for comparison. **(A)** The lower LWE is more severe than the upper LWE in this patient. **(B)** Similar manifestations in another patient. It is worth noting that the lower eyelid is severely stained near the lacrimal canal.

We scored the upper and lower LWE scores, respectively, to compare the difference between the upper and lower LWE in the same eye. For the upper LWE score, there were 48 (54.5%) patients in the moderate–severe group. For the lower LWE score, there were 51 (58.0%) patients in the moderate–severe group. The proportion of moderate and severe grades among the lower LWE scores seemed to be slightly higher than that among the upper LWE scores (Figure 4). According to Spearman’s analysis, the upper LWE staining score was moderately and significantly associated with the lower LWE staining score (r = 0.640, *p* < 0.001).

### 3.4 Comparison between the FTBUT and LWE as diagnostic indicators

According to the TFOS DEWS II (5), if we used LWE as an indicator of dry eye, all our patients would be diagnosed with dry eye, and if we used a FTBUT < 10 s as the indicator, 94.3% of our patients would be diagnosed with dry eye, leading to a missed diagnosis rate of 5.7%. However, based on dry eye diagnosis criteria in China (18) or the ADES (19), only 65.9 or 45.5% of our patients would be diagnosed with dry eye, and the remaining 34.1 or 54.5% would be missed.

## 4 Discussion

### 4.1 Ocular surface-related factors associated with the severity of LWE

A study reported a decrease in conjunctival goblet cell density and impaired MUC5AC production in patients with LWE (20). The basic hypothesis for the pathophysiology of LWE is the friction between the lid wiper epithelium and the ocular surface, which is mainly related to the following factors: (1) insufficient interface lubrication and friction force, (2) abnormal blinking, and (3) abnormalities in eyelid anatomy (21–23).

A study reported a negative correlation between total blinks and LLT (24). Since blinks had a negative effect on the tear film, incomplete blinking might lead to more adverse effects. First, the abnormalities of blinking include partial blinking, which reduces the stability of the tear film. Pult et al. (25) and McMonnies (26) both explained the relationship between blinking and LWE from the perspective of tribology. Pult proposed that if the brush structure of the lid wiper is abnormal, with an increase in blinking speed, the friction coefficient will be larger, and due to the increase in tear film viscosity, it will produce higher shear forces. So, we would like to describe it as “vicious circle of LWE and blinking,” that is, for patients with LWE lesions, abnormal blinking will cause more “blink-related microtrauma” (27) on the ocular surface, which further aggravates the LWE. McMonnies proposed that during the next blinking after an incomplete blink, the friction may be greatest, because after an incomplete blink or at the end of a prolonged interblink interval, the tear layer on the cornea may be the thinnest and the lubrication performance may be the worst. During this period, if another blink was carried out, it was easy to cause eye surface damage related to blinking. Maybe this can explain why in our study, the PBR in the moderate–severe group was significantly higher than that in the mild group. Thus, we need to realize the significance and conduct blink training in the early stage to enhance meibomian gland secretion and tear distribution. Some remedial approaches for blinking are useful to increase the frequency of complete blink rates. The tools we currently know are wink glasses (28), a light-emitting diode timer as a prompt (29), animation software (30), and so on.

Second, previous studies have suggested that the incomplete blinking affected the secretory function of the meibomian gland, thin LLT (31), and short FTBUTs (32). Although other research (33) showed that LWE was associated with LLT, we still believed that there are too many factors that influence the LLT, such as the diagnosis of diabetes (34). Confounding variables such as age and sex should be considered when assessing the importance of LLT (35–37). Based on our findings, we believe that a standardized database based on age and sex should be created in the future, and the quality of the lipid layer should also be taken into consideration, so that the LLT may then be utilized as a more reliable diagnostic parameter for dry eyes.

Our study found no significant difference in age between the different LWE groups. However, the influence of age on LWE is still debatable, with some academics arguing that reduced tear production and meibomian gland dropout with age may lead to higher friction, which may contribute to an increase in the prevalence of LWE (38).

With regard to the eyelid margin score, Ha et al. (39) found that lid margin abnormalities in the eyelid margin score were related to meibomian gland dropout and proposed the concept of a “focal dimple”. Their study also found that the focal dimple of the lower eyelids was greater than that of the upper eyelids. We also noticed the so-called “focal dimple” in our study, but we ignored it and did not analyze its existence. On this basis, the following studies can continue to prove the relationship between LWE and eyelid margin abnormalities or focal dimples and further explore whether a focal dimple of the upper and lower eyelids leads to an inconsistency of LWE.

Finally, our study found that the moderate–severe group had higher OSDI ratings than the mild group with upper LWE, although there were no statistically significant differences between such two groups with lower LWE. So, we want to know the sensitivity of LWE compared with other regions. Some researchers discovered that the lid wiper epithelium was more responsive than the other regions of the lid margin in terms of the OSDI score, which may be connected to its sensitivity (40, 41). Thus, if the LWE is severe, this may create pain in patients, which may explain the inconsistency of dry eye patients’ signs and symptoms that we call “pain without stain.”

### 4.2 The consistency and difference in upper and lower LWEs

Most studies have shown that the prevalence and severity of lower LWE were significantly higher than those of upper LWE, which was consistent with our study. Those studies presumed that they were associated with gravity, lower meibomian glands worse secretion function and quantity, tear osmolarity, and eyelid pressure (9).

McMonnies (8) suggested that there were many fewer blink-related excursions for the lower lid wiper than in the upper lid wiper, so it was mainly caused by the change of osmotic pressure. Other scholars have observed that the lower LWE was more serious at the nasal lacrimal puncta of the LWE. They believed that although the vertical movement of the lower eyelid was shorter, it would repeatedly carry out horizontal nasal movement in the same corneal conjunctiva area, so it had a higher horizontal shear force to incur friction-related damage than the upper eyelid (9). Moreover, because the direction of lipid secretion from the lower eyelid is opposite to the direction of gravity, this lipid secretion is more difficult than that of the upper eyelid (42). However, little research has been done on lower LWE, and more studies are needed to follow up and further refine the findings.

### 4.3 The possibility of LWE as an early diagnostic indicator

Our study found that if only FTBUT was used as the diagnostic indicator according to the criteria of TOFS DEWS II (FBUT < 10s), China (FBUT≤5 s or 5 s < FBUT≤10s with > 5 corneal spots) or ADES (FBUT < 5 s), 5.7% ~ 54.5% of the patients did not meet the diagnostic criteria. Our previous study (11) also found that LWE was present in 86.3% of patients with symptoms but with findings considered normal. In Korb’s study (3), this value was 76%. These indicate that LWE may be an indicator for the early diagnosis of dry eye. A study that enrolled 807 participants with dry eye, of whom more than 70% of participants fell into the mild-to-moderate category, also suggested that LWE could be an earlier clinical marker (43).

When it comes to the diagnostic criteria of dry eye, we found that there was no universal standard. For instance, the DEWSII adopted an FTBUT<10s, ocular surface staining, and tear osmolarity, and the ADES only adopted an FTBUT < 5 s for a dry eye diagnosis because the short-BUT type of dry eye is prevalent in Asian countries. However, this will result in some patients with dry eye having a missed early diagnosis, thereby they cannot receive early treatment and intervention.

Some research has shown that the Schirmer test has high variability and low reproducibility. Meanwhile, BUT is often influenced by age, race, lid size, temperature, and humidity, so that it has poor sensitivity and specificity, whereas LWE staining is an objective examination of staining with great reproducibility and high accuracy (44). However, due to the cumbersome and time-consuming nature of the two-dye staining method, it has not been fully validated in terms of reproducibility and accuracy and is not widely used in clinical practice for the time being. To resolve this issue, our previous study showed a positive rate of 81.2% for lissamine green staining and 85.9% for fluorescein staining, which suggests that both can be used alone as dyes for LWE (11). In addition, new software has been developed to objectively and reproducibly measure LWE after lissamine green staining, which will be more useful for wider clinical use in the future (45).

There are some limitations in our study. The LLT in this study represented only the thickness of the lipid layer, but not the quality of the lipid layer, which could be scored by a meibomian gland evaluator. In addition, the analysis of blinking in this study was limited to PBRs, but in recent years, studies of blinking patterns in patients with dry eyes have also been carried out. In the future, we can analyze whether there are differences in blinking patterns between dry eye patients and LWE patients and explore the characteristics of the blinking patterns in LWE patients. Moreover, we did not study the relationship between inflammation, mucins, tear osmolarity, and LWE, which will be the focus of further research in the future.

Overall, we should recognize dry eye in its early stage, but the current indicators, such as staining, generally appear in the middle and late stages in dry eye. Compared with it, although LWE is something that can easily be overlooked, it will be more suitable as an early sign of dry eye diagnosis and help the clinician explain some of the symptoms when all other ocular surface assessments look normal, if LWE is checked and detected. This is an important point as these patients may be categorized as “pain without stain” if the lid wiper area is not checked. At the same time, once we realize that “pain without stain” patients are in the early dry eye, we can carry out drug therapy or blinking training as soon as possible to break the vicious circle of dry eye, to build a healthier ocular surface environment.

## Data Availability

The raw data supporting the conclusions of this article will be made available by the authors, without undue reservation.
